# Relationship between depressive symptoms and sleep quality and cognitive inhibition ability in prenatal pregnant women

**DOI:** 10.1186/s12888-023-04976-6

**Published:** 2023-07-20

**Authors:** Ciqing Bao, Yali Wang, Tao Le, Ling Xu, Weina Tang, Wanyun Zou, Yin Bao, Dongwu Xu, Ke Zhao

**Affiliations:** 1 Wenzhou Seventh People’s Hospital, Wenzhou, 325000 China; 2 The Affiliated Brain Hospital of Nanjing Medical University, Nanjing, 210000 China; 3 School of Mental Health, Wenzhou Medical University , Wenzhou, China; 4 Shaoxing 7th People’s Hospital, Shaoxing, China; 5 Department of Obstetrics, First Affiliated Hospital of Wenzhou Medical University, Wenzhou, 325000 China; 6 Lishui Second People’s Hospital Afliated to Wenzhou Medical University, Lishui, 323000 China; 7 The Affiliated Kangning Hospital of Wenzhou Medical University Zhejiang Provincial Clinical Research Center For Mental Disorder, Wenzhou, China

**Keywords:** Executive function, Sleep disturbance, Perinatal depressive symptoms, Stroop task

## Abstract

**Background:**

Sleep problems and cognitive changes are typical in pregnant women with depressive symptoms. However, the relationship between sleep quality and executive dysfunction remains unclear. This study aims to explore the differences in sleep quality and cognitive inhibition between pregnant women with and without depressive symptoms in the third trimester of pregnancy and investigate the correlations between sleep quality, cognitive inhibition and depressive symptoms.

**Methods:**

In the third trimester, 169 women without depressive symptoms and 88 women with depressive symptoms participated in the study. Edinburgh Postpartum Depression Questionnaire (EPDS) was used to assess depressive symptoms, and Pittsburgh Standard Sleep Quality Index Questionnaire (PSQI) was used to investigate sleep quality. The color-word Stroop task is used to evaluate cognitive inhibition.

**Results:**

Compared with women without depressive symptoms, pregnant women with depressive symptoms showed worse sleep quality and Stroop task performances (response speed and accuracy). In addition, the speed of cognitive inhibition plays a mediating role in the relationship between sleep quality and prenatal depressive symptoms.

**Conclusion:**

This research emphasizes the importance of sleep quality screening and cognitive training for depression during pregnancy and childbirth in ensuring women’s mental health during pregnancy and childbirth.

## Introduction

Pregnancy is a fragile period of psychological change for women. Increasing evidence shows that the incidence rate of depressive symptoms during pregnancy is high worldwide [1, 2]. In low-income countries, the incidence is about 33.1-34.9%, while in middle-income countries, the incidence is reported as approximately 20.1-25.2% [3]. Previous studies have shown that prenatal depression is a strong predictor of postpartum depression but is more common than postpartum depression [4]. Prenatal depression manifests mainly as low mood, pessimism, decreased interest and motivation, restlessness, sleep disturbance and weight loss, and even suicidal ideation. Prenatal depression has been shown to impact maternal and infant health negatively, being correlated to outcomes such as premature birth [5, 6], obstetric complications [7], interference with mother-infant contact [8] and increased risk of postpartum depression [9, 10]. Many risk factors of prenatal depression have been identified, including life stress, previous depression, lack of social support, and sleep disturbances [11, 12]. Sleep disturbances are an essential risk factor for prenatal depression, and treatment of prenatal insomnia may prevent the onset of prenatal depression and anxiety [11, 13].

It is widespread for women to suffer from sleep disturbances during pregnancy [14, 15]. Recent studies have reported that women’s insomnia can rise to 60% during pregnancy [16]. As pregnancy progresses, the frequency and duration of sleep disorders will increase [17, 18]. Several studies have examined the relationship between sleep and depression during pregnancy [19–21], reporting a significant cross-sectional and vertical association between sleep, depression and anxiety [19, 21, 22]. A meta-analysis of insomnia and maternal depression also found that maternal insomnia symptoms (i.e., difficulty falling asleep and/or staying asleep), poor sleep quality, and night waking were positively correlated with depression [23].

The common risk factors associated with depression and sleep disturbances are cognition. At the cognitive level, a recent study found that fragmented sleep may lead to cognitive impairment in pregnant women in the third trimester of pregnancy [24]. Healthy people with acute or chronic sleep disorders can also experience impaired cognitive function, especially executive control, including decision-making, working memory, and attention [25, 26]. A recent study reported that sleep deprivation impaired executive control (demonstrated by longer response time and more errors during the Stroop and go/no-go tests) [27]. Individuals with sleep problems have a 1.65 times higher risk of cognitive impairment than individuals without sleep issues [28]. It is agreed that sleep affects cognition, and a correlation exists between cognition and depressive symptoms. In previous studies of executive function in depressed patients, compared with healthy controls, depressed patients showed deficits in executive control function, reflected primarily in slower information processing and requiring more cognitive effort [29]. However, few studies have explored the executive function associated with prenatal depression. Sleep quality and cognitive inhibition may interact and play an important role in forming and developing of perinatal depression.

Consequently, this study aims to consider the differences in sleep quality and cognitive inhibition for women with or without depressive symptoms in the third trimester of pregnancy and investigate the relationship between prenatal depression, cognitive inhibition, and sleep quality. The hypothesis being addressed is that pregnant women with prenatal depressive symptoms would experience more sleep disturbance and cognitive inhibition deficits. Additionally, whether cognitive inhibition mediates a relationship between sleep quality and prenatal depressive symptoms will be explored.

## Methods

### Participants

This study is part of a more extensive longitudinal study of maternal mental health conducted at the First Affiliated Hospital of Wenzhou Medical University between February 2017 to June 2019. From December 2017 to June 2018, 400 pregnant women signed informed consent forms and only 271 women completed the scale and the classic Stroop task. After excluding 10 individuals with incomplete data and 4 individuals with invalid data, the remaining 257 individuals were included in the study, with a response rate of 64.25%. According to the testing using G-power software (expected significance P<0.05; expected test force 1-β = 0.8), the final data met the sample size standard. This study was recruited through recruitment advertisements in obstetric clinics. Participants were enrolled in the third trimester of pregnancy, and the investigator only further screened women for eligibility after obtaining oral consent. Participants were able to withdraw from the study at any time. The inclusion criteria are: (1) Adult (≥ 18 years old); (2) Late pregnancy (28 weeks to 40 weeks); (3) In the research hospital, regular inspections and delivery;(4) Signing informed consent, and(5) No sleep problems before pregnancy. The study was undertaken by psychiatry graduate students and physicians who had undergone consistent training before the experiment began. The data collected includes all pregnant women’s demographic and clinical information in face-to-face interviews via standardized questionnaires, including age, height, weight, nation, residential area, education years, family monthly income, medical history, and psychiatric disorder history. We excluded women with severe pregnancy complications or any mental/cognitive problems that would prevent them from completing the survey, including pregnant women with a history of prenatal depression, other mental illnesses and mental retardation.

This study protocol followed the ethical guidelines of the 2013 Declaration of Helsinki and was approved by the Medical Ethics Committee of the Wenzhou Medical University.

### Measurements

#### Sleep quality

The Pittsburgh Sleep Quality Index (PSQI) [30] was used to assess sleep quality over the previous month. The maximum total score of the PSQI is 21 points. Nineteen individual items generate seven component scores (range 0–3, with higher scores indicating worse sleep): subjective sleep quality, sleep latency, sleep duration, habitual sleep efficiency, sleep disturbances, use of sleeping medication, and daytime dysfunction. This scale has internal consistency, test-retest reliability, and validity [31]. A total score of PSQI > 5 indicates clinically significant sleep disturbance [32]. In this study, Cronbach’s α coefficient is 0.74.

#### Depressive symptoms

In western countries, the Edinburgh Postnatal Depression Scale(EPDS) [33] is a widely used depression assessment scale. And it is believed to be effective in multiple cultures during pregnancy and postpartum [34–37]. It has 10 items testing: mood, pleasure, guilt, anxiety, fear, insomnia, ability to cope, sadness, crying, and self-injury. In accordance with the severity of the relevant symptom, each item is divided into 0–3 points and the total score ranges from 0 to 30 points. The scale has good reliability and validity in populations in mainland China [38]. In this experiment, a total score of > 9 was classified as depressive symptoms [39], and Cronbach’s α coefficient was 0.77.

#### Cognitive inhibition

The color word Stroop task [40] is a classic experimental paradigm in behavioral neuroscience in clinical and research settings. The classic color word Stroop task [40] is used to study the ability to measure cognitive impairment inhibition, selective attention, memory, and cognitive flexibility. E-Prime Psychology Software Tools design the Stroop Mission. When the nouns appearing were color names that were visually displayed in different colors, the subjects were strongly interfered with by the word reading on the naming of the color, called the Stroop interference effect. The experiment used in this study is called the Stroop word color interference test. The experiment gives a single word on a computer screen. These word stimuli consisted of a random presentation of four-color names (red, green, yellow, or blue) in one of these four colors. The words stand alone, on a black background, with the computer screen 40 cm directly in front of the subject’s face. Ninety-six experiments were performed, with 48 trials for each condition. The words were displayed for 1300 msec with an interstimulus interval of 350 msec (1300 msec on, 350 msec off). In the congruent condition, the color in which the word was presented matched the color name (e.g., the word red was displayed in red color). In the incongruent condition, the color of the word presented did not match the color name (e.g., the word red was displayed in green). No color names or presentation colors were ever repeated consecutively. In both cases, subjects were instructed to distinguish the colors and meanings of the words as quickly as possible. If they were consistent, press the “J” key on the keyboard, and if not, press “F” on the keyboard. The computer automatically records the press response time and the correctness of the results. There are eight words after the task begins to give the subject a pre-test to facilitate understanding of the principle, not counting the total score.

### Statistical analysis

The Statistical Package for the Social Sciences (SPSS) version 22.0 was used for statistical analysis. For analysis of the prenatal data, participants were divided into two groups based on their EPDS score: those who scored ten or above comprised the depressive-symptom group (n = 88), and those who scored nine or below comprised the nondepressive-symptom group (n = 169). Descriptive statistics were estimated for continuous measures using means and standard deviations, and frequencies and proportions for categorical measures. When continuous measures met distributional assumptions of normality, Student t-tests were used to test group differences. Otherwise, the non-parametric Wilcoxon test was used, while the chi-square tests were performed for categorical measures.

Pearson correlation analysis explored the relationships between sleep quality, cognitive inhibition and prenatal depressive symptoms. Two mediation models were tested using the PROCESS macro, founded on the principles by Hayes and Preacher [41, 42]. In the mediation model, path a is the association between X and M, path b represents the association between M and Y and the indirect effect is the product of path a times path b. Path c represents the total effect of the relationship between X and Y ignoring the mediator (path c = indirect effect + direct effect). In the two models, sleep quality was defined as the predictor variable (X), prenatal depressive symptoms were defined as outcome variables (Y) and cognitive inhibition speed/accuracy (response time/accuracy in Stroop task) were entered as the mediator variable (M), respectively. The direct effect of sleep quality on prenatal depressive symptoms (path c’) and its indirect effect through the mediator variable (response inhibition speed/accuracy) were both tested. An estimation of the proportion mediated (PM) is reported, which indicates how much of the total effect operates through the mediator [43]. A bootstrapping method based on 5000 samples and a confidence interval of 95% was used to quantify effects.

## Results

### Demographic and clinical variables

The demographic characteristics of the sample are shown in Table 1. All the subjects are of Han nationality. There is no statistical difference between the two groups in age, body mass index (BMI), years of education, residence, monthly family income, smoking, drinking and exercise habits(*p* > 0.05).

**Table 1:** Comparisons of demographic characteristics

	Nondepressive-symptom group(n = 169)	Depressive-symptom group (n = 88)	*t*/*χ*^*2*^	*p*
Gestational weeks	35.60 ± 1.98	35.69 ± 3.11	0.26	0.79
Age (year)	28.67 ± 4.03	28.73 ± 3.71	0.11	0.91
BMI (kg/m^2^)	25.12 ± 2.51	25.18 ± 2.97	0.17	0.86
Education (year)	13.81 ± 2.27	13.25 ± 2.55	1.79	0.08
Number of abortion	0.53 ± 0.89	0.57 ± 0.87	0.31	0.76
Number of children	0.43 ± 0.52	0.53 ± 0.53	1.58	0.12
Residence			0.37	0.54
Town	70(41.4%)	33(37.5%)		
Countryside	99(58.6%)	55(62.5%)		
Monthly family income (RMB)			0.45	0.80
<5000	38(22.5%)	23(26.1%)		
5000–10,000	72(42.6%)	35(39.8%)		
>10,000	59(34.9%)	30(34.1%)		
Currently drinking, %	2(1.2%)	3(3.4%)	0.56	0.34
Currently smoking, %	0(0%)	1(1.1%)	0.11	0.34
Long-term exercise habits			0.50	0.48
No	151(89.3%)	76(86.4%)		
Yes	18(10.7%)	12(13.6%)		

The characteristics of depressive symptoms and sleep are shown in Table 2. As expected, the total score of the Edinburgh Postpartum Depression Scale (*t* = 19.30, *p* < 0.001) and the Pittsburgh Sleep Quality Index (*t* = 5.51, *p* < 0.001) of women with depressive symptoms are higher than those of women without depressive symptoms. In the third trimester of pregnancy, compared to women without depressive symptoms, women with depressive symptoms have worse subjective sleep quality (*t* = 4.23, *p* < 0.001), longer sleep latency *(t* = 3.91, *p* < 0.001), shorter sleep duration (*t* = 1.98, *p* = 0.050), more severe sleep disturbances (*t* = 6.17, *p* < 0.001), and more daytime dysfunction (*t* = 5.19, *p* < 0.001). However, there is no difference between the two groups in sleep efficiency (*t* = 0.90, *p* = 0.370). In this study, none of the pregnant women took any sleep medication.

**Table 2:** Comparisons the results of self-report scales

	Nondepressive-symptom group(n = 169)	Depressive-symptom group (n = 88)	*t*	*p*
EPDS	6.02 ± 2.05	12.28 ± 2.67	19.30	< 0.001
PSQI (total)	6.06 ± 2.98	8.38 ± 3.57	5.51	< 0.001
Subjective sleep quality	1.23 ± 0.60	1.59 ± 0.67	4.23	< 0.001
Sleep latency	1.27 ± 0.92	1.75 ± 0.97	3.91	< 0.001
Sleep duration	0.33 ± 0.80	0.58 ± 1.06	1.98	0.05
Habitual sleep efficiency	0.85 ± 1.03	0.98 ± 1.11	0.90	0.37
Sleep disturbances	1.21 ± 0.51	1.65 ± 0.55	6.17	< 0.001
Use of sleeping medication	0	0	-	-
Daytime dysfunction	1.17 ± 0.94	1.81 ± 0.93	5.19	< 0.001

***Note.*** EPDS = the total score of the Edinburgh Postnatal Depression Scale; PSQI = the total score of the Pittsburgh Sleep Quality Index

**Fig. 1:**
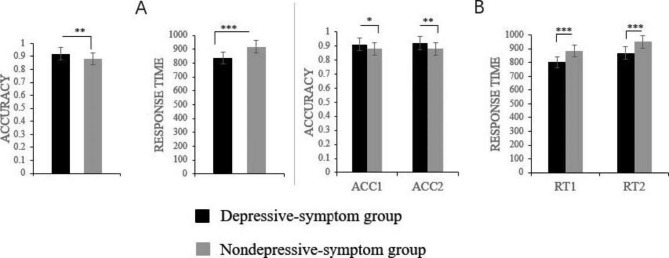
Performances of Stroop task. Figure 1 **A** shows the Depressive-symptom group performed significantly worse than the Nondepressive-symptom group in total cognitive inhibition response time and accuracy. Figure 1**B** shows that the Depressive-symptom group had significantly worse cognitive speed/accuracy than the in both conditions. ACC1 = accuracy in the color and word inconsistencies; ACC2 = accuracy in the color and word consistencies; RT1 = response time in the color and word inconsistencies; RT2 = response time in the color and word consistencies. * p < 0.05, ** p < 0.01, *** p < 0.001

The results of the independent sample t-test showed that the difference in total cognitive inhibition response time (*t* = 5.232, *p* < 0.001) and accuracy (*t* = 3.183, *p* < 0.01) between the two groups is also statistically significant. In the color and word inconsistencies condition, women with depressive symptoms need a longer time to respond (*t* = 4.923, *p* < 0.001) and have a lower response accuracy (*t* = 2.756, *p* = 0.007). The same is true for the response time (*t* = 5.232, *p* < 0.001) and accuracy (*t* = 2.551, *p* = 0.011) of the two groups in the color and word consistency condition. More details can be seen in Fig. 1.

### Association between prenatal sleep quality, cognitive inhibition, and prenatal depressive symptoms

Pearson correlation analysis showed that mean response time in the Stroop task was significantly positively correlated with the Pittsburgh Sleep Quality Index score and the total score of the Edinburgh Postnatal Depression Scale (*r* = 0.142, *p* = 0.032; *r* = 0.223, *p* < 0.001). Furthermore, the Pittsburgh Sleep Quality Index score positively correlates with the Edinburgh Postnatal Depression Scale (*r* = 0.381, *p* < 0.001). After a Bonferroni correction, the relationships between the total score of EPDS with mean response time and the total score of the EPDS with the score of PSQI remains significant (see Table 3).

**Table 3:** Correlation analysis

	1	2	3	4	5	6	7	8	9	10
1.RT	-									
2.ACC	-0.158^*^	-								
3.EPDS	0.223^***b^	-0.116	-							
4.PSQI	0.142^*^	-0.022	0.381^***b^	-						
5.Subjective Sleep quality	0.100	-0.044	0.339^***b^	0.725^***b^	-					
6.Sleep latency	0.101	-0.052	0.321^***b^	0.668^***b^	0.442^***b^	-				
7.Sleep duration	0.158^*^	-0.032	0.101	0.713^***b^	0.452^***b^	0.306^***b^	-			
8.Habitual sleep efficiency	0.030	0.064	0.061	0.689^***b^	0.271^***b^	0.364^***b^	0.576^***b^	-		
9.Sleep disturbances	0.098	-0.024	0.381^***b^	0.552^***b^	0.451^***b^	0.307^***b^	0.159^*^	0.181^**^	-	
10.Daytime dysfunction	0.077	-0.021	0.385^***b^	0.571^***b^	0.429^***b^	0.165^**^	0.207^***^	0.110	0.367^***b^	-

***Note.*** RT = mean response time in the stroop task; ACC = mean accuracy in the stroop task; EPDS = the total score of the Edinburgh Postnatal Depression Scale; PSQI = the total score of the Pittsburgh Sleep Quality Index. ^*^p < 0.05, ^**^p < 0.01, ^***^p < 0.001. b represents significance level at *p*<0.05 with Bonferroni correction

### The mediating effect of cognitive inhibition speed in sleep quality and prenatal depressive symptoms

**Fig. 2:**
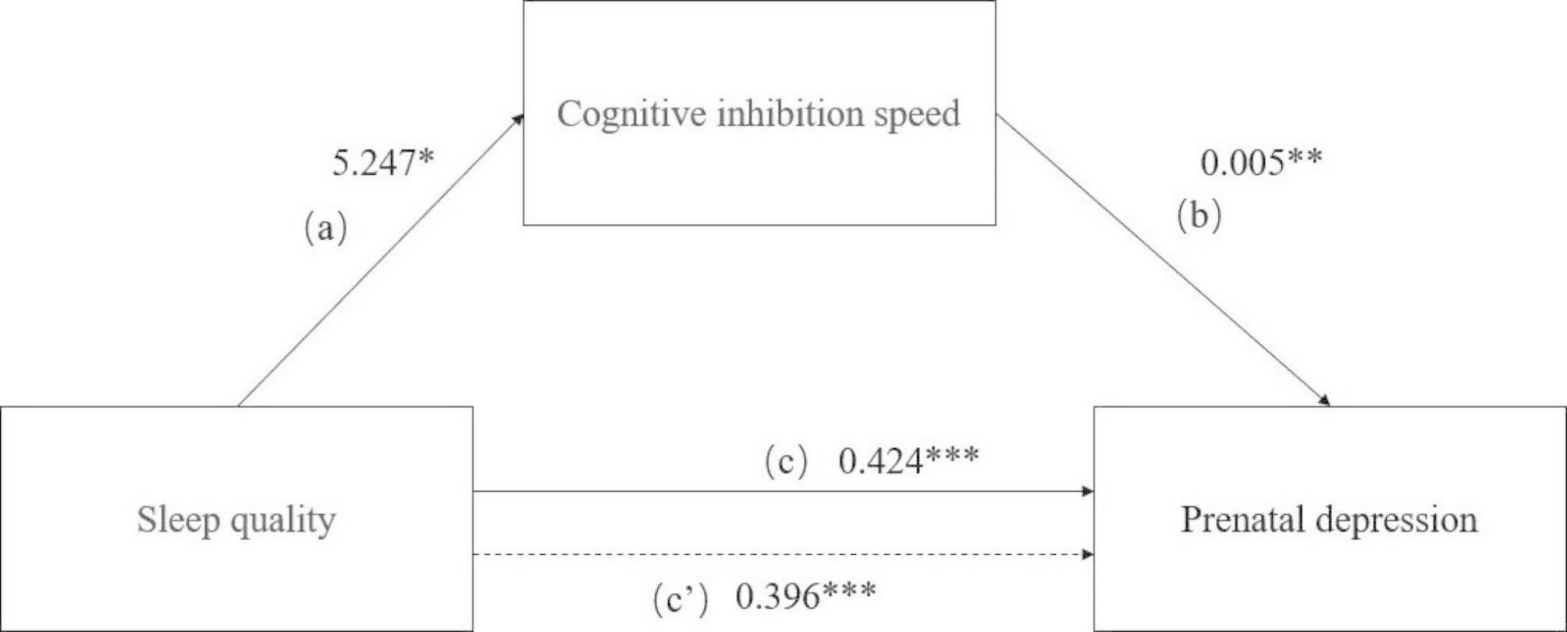
Results of mediation analysis. The figure illustrates the mediating role of cognitive inhibition speed in the relationship between sleep quality and prenatal depressive symptoms. Path c represents the total effect of sleep quality on prenatal depressive symptoms. Path c’ represents the direct effect of sleep quality on prenatal depressive symptoms without considering the mediation of cognitive inhibition speed. Cognitive inhibition speed = mean response time in the Stroop task; Prenatal depressive symptoms = the total score of the Edinburgh Postnatal Depression Scale; Sleep quality = the total score of the Pittsburgh Sleep Quality Index. ^*^p < 0.05, ^**^p < 0.01, ^***^p < 0.001

Regression analyses found a significant mediating role of cognitive inhibition speed in the relationship between sleep quality and prenatal depressive symptoms. (path a = 5.247, *se* = 2.298, *p* = 0.023; path b = 0.005, *se* = 0.064, p < 0.001; path c = 0.424, *se* = 0.064, *p* < 0.001; path c’ = 0.396, *se* = 0.064, *p* < 0.001; indirect effect = 0.028; PM = 6.604%; see Fig. 2). The mediating effect of cognitive inhibition accuracy does not hold.

## Discussion

This study explores the relationship between sleep quality, prenatal depressive symptoms, and cognitive inhibition in pregnant women. In general, during the third trimester, the results indicate that pregnant women with depressive symptoms are more likely to have sleep problems than those without depressive symptoms. Additionally, pregnant women with depressive symptoms have cognitive inhibition defects. Across all subjects, women with depressive symptoms correlate positively with decreased sleep quality and slower cognitive inhibition speeds. Further analysis shows that the speed of cognitive inhibition is crucial in the relationship between sleep quality and prenatal depressive symptoms.

The third trimester of pregnancy is the most unstable period of sleep throughout pregnancy [44, 45]. The sleep time of depressed women in the second half of pregnancy was significantly more dispersed, showing a longer sleep latency and longer awake time at night [44]. Another study found that depressed pregnant women woke up more often at night and spent more time trying to fall asleep [45]. Such findings are consistent with the current study’s data. In the third trimester, compared with women without depressive symptoms, women with depressive symptoms have worse sleep quality, manifested in worse subjective sleep quality, longer sleep latency, shorter sleep time, and more sleep disorders. At the same time, the daytime function of women with depressive symptoms decreases. However, no significant difference in sleep efficiency between the two groups was found, at variance to previous research results [44], and may be related to the inclusion criteria of depressed subjects in this study.

In the Color-Word Stroop task, the response speed of pregnant women with depressive symptoms is significantly slower than that of pregnant women without depressive symptoms, and the accuracy is reduced. Such an outcome may be related to the impaired executive function of pregnant women with depressive symptoms, which indicates that pregnant women with depressive symptoms have difficulty in the ability of cognitive inhibition. There are few studies on Stroop tasks of women experiencing prenatal depression. In the broader depressed population, depressed patients require more cognitive effort and longer cognitive processing time when performing executive functioning tasks [29]. This study illustrates the characteristics of cognitive inhibition in patients with depressive symptoms and further supports the existence of executive function deficits in women with depressive symptoms during pregnancy.

Insomnia and depression are stress-related disorders commonly occurring in women during pregnancy [46]. The current study found that a direct effect of sleep disturbance on prenatal depressive symptoms and an indirect effect on prenatal depressive symptoms through cognitive inhibition speed. Results from our study show that a higher PSQI score correlates to a higher prenatal EPDS score. This outcome is consistent with those from the Finn Brain Birth cohort study [47] and the Anhui province of China study [11], which found that sleep disturbance in the third trimester of pregnancy is positively correlated with prenatal depression.

Impaired cognitive function has been reported to be found in the general population with sleep problems as well as in pregnant women [24, 27], and MRI evidence suggests that working memory impairment due to sleep deprivation is associated with reduced activity in the left parietal and prefrontal regions [48]. At the neurocognitive and molecular level, depression is currently described as a disorder of impaired cognitive flexibility and a failure of neuroplasticity [49, 50], including neuronal atrophy and synaptic inhibition in the medial prefrontal cortex and hippocampus [51, 52]. This study found that sleep quality (PSQI total score) is positively correlated with the speed of cognitive inhibition, which may be due to deterioration in sustained attentional capacity, working memory, and learning capacity caused by chronic sleep disturbance [53, 54].

This study also found that impaired cognition is positively associated with depressive symptoms, consistent with the cognitive neuropsychological model of depression, where impaired cognition assumes an essential role in the onset and maintenance of depression [55]. In summary, depression is reliably associated with impaired cognition [56], but the causal relationship remains to be explored in the future [57]. The current study supports that impaired cognition may contribute to the occurrence and development of depressive symptoms. Future studies could investigate whether frequent cognitive function tests and timely interventions could mitigate subsequent depression if performed early during the uncomplicated or depressed period.

Finally, the accuracy of cognitive inhibition did not mediate the association between sleep quality and depressive symptoms during pregnancy. However, there is a decrease in the accuracy of cognitive inhibition in women with depressive symptoms. A meta-analysis of studies on the relationship between executive function and sleep deprivation reported that episodic short-term sleep deprivation decreases attentional response speed but not accuracy [58], which is consistent with data from the current study. Further studies are needed to explore the mechanism of the impairment of cognitive inhibition accuracy of women with depressive symptoms during pregnancy.

To reduce the risk of perinatal depressive symptoms, improving pregnant women’s sleep quality and executive functions is essential. Firstly, women during pregnancy should be encouraged to carry out comprehensive sleep screening, identify sleep problems early, and initiate interventions. Secondly, studies at the electrophysiological level have found that restoring sleep reverses impaired response inhibition due to sleep restriction [59]. Hospitals must carry out comprehensive sleep management plans for pregnant women (such as sleep health education [60]) for self-regulation. Finally, based on improving sleep quality, planned executive function training (such as neurofeedback training [61]) can reduce the adverse effects of sleep disturbance during perinatal depression.

There are several limitations in the current study. Firstly, due to the nature of cross-sectional studies and the small sample size, as well as the fact that they were not conducted in multiple centers, the dynamic relationship between sleep and executive function in perinatal depressive patients during pregnancy is uncertain. Therefore, whether the defect of sleep quality and response inhibition ability arises from state or characteristic damage is not evident. Secondly, this study did not have non-pregnant women as control subjects, so differences in sleep quality and cognitive inhibition between pregnant and non-pregnant women could not be ascertained. Thirdly, only EPDS was used to screen prenatal depressive symptoms, so structured interviews were not conducted to diagnose prenatal depression. Finally, due to the absence of brain imaging, whether any neuroanatomical/functional changes occurred in the participant’s brains is unknown.

## Conclusion

In summary, the quality of sleep and the speed of cognitive inhibition during pregnancy are correlated with prenatal depressive symptoms. Moreover, the speed of cognitive inhibition plays a mediating role in the relationship between sleep quality and prenatal depressive symptoms. The data suggest a significant role for sleep quality screening and intervention in preventing maternal depression, emphasizing the importance of maternal depressive symptom screening and executive function training to support and ensure pregnant women’s mental health.

## Data Availability

The datasets generated during the current study are not publicly available due to the subjects’ privacy but are available from the corresponding author on reasonable request.

## Abbreviations

EPDS: Edinburgh Postpartum Depression Questionnaire
PSQI: Pittsburgh Standard Sleep Quality Index
HPA: Hypothalamus pituitary adrenal
BMI: Body mass index
